# Current Understanding of Cardiovascular Calcification in Patients with Chronic Kidney Disease

**DOI:** 10.3390/ijms251810225

**Published:** 2024-09-23

**Authors:** Sijie Chen, Rining Tang, Bicheng Liu

**Affiliations:** Institute of Nephrology, Zhongda Hospital, School of Medicine, Southeast University, Nanjing 210009, China; chensjseu@seu.edu.cn (S.C.); tangrn77@seu.edu.cn (R.T.)

**Keywords:** chronic kidney disease, cardiovascular calcification, mechanism, signaling pathway

## Abstract

The burden of chronic kidney disease (CKD) is increasing, posing a serious threat to human health. Cardiovascular calcification (CVC) is one of the most common manifestations of CKD, which significantly influences the morbidity and mortality of patients. The manifestation of CVC is an unusual accumulation of mineral substances containing calcium and phosphate. The main component is hydroxyapatite. Many cells are involved in this process, such as smooth muscle cells (SMCs) and endothelial cells. CVC is an osteogenic process initiated by complex mechanisms such as metabolic disorders of calcium and phosphorus minerals, inflammation, extracellular vesicles, autophagy, and micro-RNAs with a variety of signaling pathways like Notch, STAT, and JAK. Although drug therapy and dialysis technology continue to advance, the survival time and quality of life of CVC patients still face challenges. Therefore, early diagnosis and prevention of CKD-related CVC, reducing its mortality rate, and improving patients’ quality of life have become urgent issues in the field of public health. In this review, we try to summarize the state-of-the-art understanding of the progression of CVC and hope that it will help in the prevention and treatment of CVC in CKD.

## 1. Introduction

Chronic kidney disease (CKD) is a serious disease that involves damage to structure and/or function of kidneys via multiple causes, and it is a great threat to global public health. According to epidemiological data, the current global prevalence of CKD is about 9.5%, with a continuous annual increase [1]. There are approximately 130 million patients with CKD in China [2]. In 2017, about 1.2 million patients with CKD died globally, and another 1.4 million died of cardiovascular diseases (CVDs) related to renal failure, indicating that CVDs are an important cause of death in patients with CKD [3]. CKD causes a high incidence of cardiovascular calcification (CVC). Patients with CKD are four times more susceptible to CVC than healthy individuals [4]. CVC is an important component and prognostic factor of CVD [5]. At present, the pathogenesis of CVC in patients with CKD is still not sufficiently clear. Thus, an in-depth understanding of the pathogenesis of CVC will assist in the prevention and treatment of cardiovascular complications in patients with CKD and subsequently improve the patients’ outcomes.

**Figure 1:**
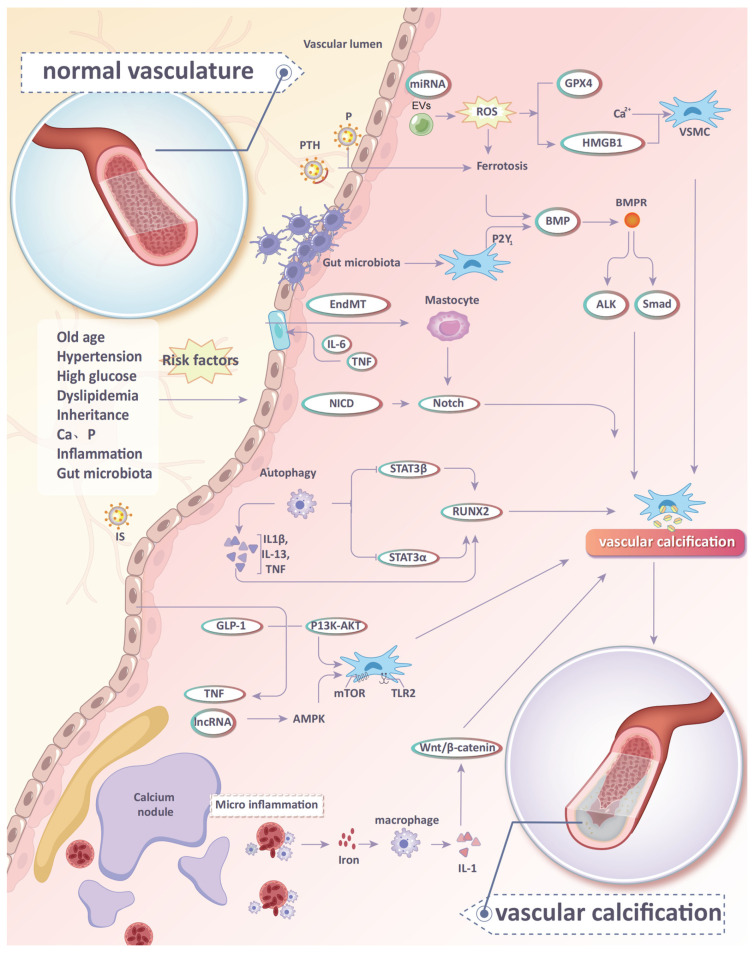
The mechanism diagram of CVC. P: phosphorus, PTH: parathyroidhormone, IS: Indoxylsulfate, VSMCs: vascular smooth muscle cells, miRNAs: micro-RNAs, EVs: extracellular vesicles, Runx2: runt-related transcription factor 2, BMP2: bone morphogenetic protein, TNF: tumor necrosis factor, IL: interleukin, ROS: reactive oxygen species, EndMT: endothelial-to-mesenchymal transition, STAT: transcriptional activation factor, NICD: Notch intracellular domain.

Vascular calcification (VC) manifests as an unusual accumulation of mineral substances containing calcium and phosphate, mainly in the form of hydroxyapatite. According to the major calcifying site of the vascular wall, VC typically involves endometrial calcification and media calcification, which are based on different mechanisms [6]. Endometrial calcification is common in individuals with arteriosclerosis, whereas media calcification is common in patients with CKD and diabetes. The main manifestation is a change in the phenotype of smooth muscle cells (SMCs). Specifically, SMCs transdifferentiate from the contractile phenotype to the osteoblastic phenotype. At the same time, the expression of contractile phenotypic marker genes such as smooth muscle 22 alpha (SM22α) and α-smooth muscle actin (α-SMA) decreases, while the expression of bone morphogenetic protein (BMP2), osteopontin (OPN), runt-related transcription factor 2 (Runx2), and other osteogenesis markers increases [7]. This process is dominated by SMCs, but a variety of other cell types, such as endothelial cells (ECs), macrophages, circulating progenitors, pericytes, and vascular outer membrane fibroblasts, actively participate in the process. In addition, a variety of risk factors, molecular mechanisms, and signaling pathways constitute a complex signaling network for regulation (Figure 1) [8].

## 2. Risk Factors of CVC

The traditional risk factors of CKD include old age, hypertension, diabetes, dyslipidemia, heredity, etc. These factors will lead to the occurrence and development of CVC. Hypertension is a common complication of CKD, which can lead to kidney damage and promote the progression of CKD. Hypertension can also cause hemodynamic disorders, leading to damage to target organs such as the heart, brain, and surrounding blood vessels, especially exacerbating the progression of CVC [9]. CVC is a common vascular complication of diabetes, and the abnormal metabolism caused by hyperglycemia is the pathological basis of cardiovascular damage. Persistent hyperglycemia can lead to an increase in glycation end products (Advanced Glycosylation End, AGEs) in the body, which promote the occurrence and development of CVC through AGEs’ receptors [10]. It was found that NF90 mediated CVC in diabetes through the FBXW7-AGER1-AGES pathway [11]. Dyslipidemia is also an important factor to promote the progress of CKD, and also a major risk factor to mediate cardiovascular and cerebrovascular disease, renal atherosclerosis, and target organ damage in CKD. Elevated blood lipid components and abnormal lipid components such as oxidized low-density lipoprotein and glycosylated low-density lipoprotein can damage vascular tissue, promoting the production of the extracellular matrix [12]. Here, we will mainly focus on the non-traditional risk factors which are specifically involved in CVC in CKD patients.

### 2.1. Disturbance of Calcium and Phosphorus Metabolism

Disorder of calcium and phosphorus metabolism is universal in patients with CKD. Under normal circumstances, fibroblast growth factor 23 (FGF23) and the FGF23 receptor inhibit phosphate absorption, promoting its excretion through urine. In patients with CKD, the level of FGF23 continues to increase, but phosphate excretion is inhibited, while serum calcium and phosphorus levels increase with calcium and phosphorus products increasing accordingly, thereby destroying the ectopic calcification inhibition mechanism of vascular SMCs (VSMCs). Cytokines play an important role in this process by inducing osteogenic transdifferentiation and hydroxyapatite deposition, finally causing vascular media calcification [13]. In vitro experiments have shown that osteogenic factors BMP2 and Runx2 in VSMCs are upregulated, and that calcification is intensified under high-phosphorus environment [14]. At the same time, the continuous state of high phosphorus and low calcium stimulates parathyroid hormone (PTH), which regulates calcium and phosphorus levels. However, excessive PTH leads to secondary hyperparathyroidism (SHPT), further aggravating the imbalance of calcium and phosphorus metabolism, forming a vicious cycle [15]. In addition, the disturbance of calcium and phosphorus metabolism can lead to an increased inflammation level in CKD, which promotes the occurrence of VC, while inhibition of the inflammatory factors can alleviate osteogenic transdifferentiation and relieve the process of VC [16]. The above evidence suggests that the disordered metabolism of calcium and phosphorus drives CVC. Hence, regulating this disturbance may become a potential therapeutic target for CVC.

### 2.2. Inflammation

Persistent micro-inflammation is an important pathophysiological state of CKD. It increases cytokines that promote inflammation, such as interleukins (ILs), upregulating proinflammatory factors of the vascular wall and osteogenic transdifferentiation factors, thereby causing CVC. Inflammation is triggered before osteogenic transdifferentiation of SMCs and the release of calcified stromal vesicles [17]. Previous studies have shown that macrophages derived from monocytes are extensively involved in the entire process of VC, from micro-calcification to osseus metaplasia, producing proinflammatory factors, inducing oxidative stress damage, and mediating bone-like transformation and calcification of blood vessels and heart valve cells [18]. Crystallization of calcium and phosphorus promotes the polarization of M0 macrophages to the M1 type and recruits inflammatory immune cells to the calcified site. It has been shown that macrophages treated with IL-4 or IL-13 promote arteriosclerosis as a result of stimulation by transforming growth factor-β (TGF-β). Nucleotide-binding oligomerization domain 2 (NOD2) belongs to the nucleotide combined with the oligomeric receptor family. NOD2 has been shown to induce inflammation and exacerbate kidney injury [17]. Moreover, the deficiency in NOD2 can promote proliferation, migration, and neovascularization of VSMCs following vascular injury, as well as the development of atherosclerosis [19], which can progress to VC. In the intact state of CKD, the activation of NF-κB signaling pathways induces osteogenic markers, ultimately leading to the occurrence and development of CVD. Agents that selectively inhibit NF-κB signaling may become new candidates for the treatment and prevention of arterial medial calcification in CKD [20]. In summary, there are multiple levels of evidence supporting the crucial role of inflammation in CVC, and the specific mechanism is still being explored.

### 2.3. High Glucose

High glucose promotes CVC. High glucose induces nonenzymatic glycation reactions, which contribute to the accumulation of AGEs that interact with surface receptors of ECs, resulting in cell damage. Damaged endothelial cells acquire osteogenic differentiation potential through endothelial mesenchymal transition or generate sustained low-intensity immune inflammatory activation and cytokine release, inducing VSMC osteogenic differentiation, affecting the homeostasis of vascular walls and ultimately leading to the formation of calcified plaques. Research has shown that high glucose can upregulate the expression of bone morphogenetic protein, an osteogenic marker such as OPN, while increasing arterial calcium content and promoting the deposition of large amounts of calcium salts in blood vessels [21]. This suggests that metabolic disorders in a high glucose state can trigger VSMC phenotype transition and vascular calcification [22]. SGLT2 inhibitors improve CVC by inhibiting the upregulation of endoplasmic reticulum stress-dependent TXNDC5 and promoting degradation of osteogenic-related factors such as Runx2, indicating potential benefits of SGLT2 inhibitors in the prevention and treatment of CVC. A high glucose environment can lead to an increase in the expression of the osteogenic marker OPN in VSMCs, which is involved in the development of vascular calcification [23]. High glucose levels can lead to the occurrence of vascular calcification.

### 2.4. Gut Microbiota

Gut microbiota affects human health. Dysfunction of gut microbiota enhances the risk of CVDs [24]. Uremic toxins accumulated in the intestine can affect the intestinal microenvironment, causing the abnormal metabolism of amino acids and choline, further promoting the levels of enteric-borne toxins. With the deterioration of renal function, renal clearance function is also impaired. Uremic toxin accumulation and intestinal barrier damage are further aggravated, forming a vicious cycle [25]. Many gut microbiota metabolites are closely related to CVC in CKD. For example, trimethylamine oxide (TMAO) can activate the NLRP3 and NF-κB signaling pathway, promoting the osteogenic differentiation of SMCs and the development of CVC in CKD rats. Indoxylsulfate (IS) and p-cresylsulfate (PCS) inhibit proliferation and repair of EC damage and promote the release of EC pro-calcification vesicles [26]. IS can also induce osteogenic differentiation and apoptosis of VSMCs via Notch signaling, thereby promoting calcification. In vivo studies have shown that IS promotes the upregulation of aortic osteogenic markers and the deposition of calcified crystals. Inhibition of intestinal absorption of microbial metabolites, such as via the use of oral activated carbon adsorbent AST-120, can alleviate aortic calcification in patients with CKD [27]. Intestinal barrier dysfunction and gut microbiota dysregulation and translocation are associated with systemic inflammatory status in CKD, oxidative stress, and deterioration of renal function, which all accelerate the development of VC. These findings suggest that dysregulation of gut microbiota promotes CVC. However, there is still a lack of research on its function in CVC. It is necessary to deeply explore gut microbiota to provide ideas for treatment in the future.

## 3. Mechanisms of CVC in CKD

### 3.1. Autophagy

Autophagy is a process of intracellular degradation of lysosomal catabolites and organelles, which can resist hypoxia, inflammation, oxidative stress, and other stimuli. Mitochondrial autophagy is a process of clearing dysfunctional or damaged mitochondria via specific molecular mechanisms, and it contributes to maintaining homeostasis for cells and organisms. Disequilibrium of autophagy contributes to many diseases, such as CVDs, tumors, and neurodegenerative diseases [28]. Autophagy is closely related to calcified CVD. In calcified tissues, mitochondrial respiratory function is impaired, ATP production is inhibited, and reactive oxygen species (ROS) are produced, resulting in increased cell death and Ca^2+^ imbalance, driving the calcification process [29]. M1 macrophages can promote VC of SMCs via affecting autophagy through TSNA-5006S, and targeted inhibition of this factor can reduce osteogenic markers and alleviate the occurrence and development of calcification. High phosphorus induces ROS production in vitro and in vivo and promotes autophagy [30]. As a result, the release of calcified vesicles is reduced. Increased levels of autophagy are also observed in mice fed high-phosphorus diets. Inhibition of autophagy exacerbates VSMCs’ calcification, whereas further induction of autophagy with rapamycin inhibits VSMCs’ osteogenic transformation and alleviates CVC [31]. Hence, autophagy changes the phenotype and promotes osteogenesis of VSMCs. However, the complex interplay caused by autophagy needs more research.

### 3.2. Ferroptosis

Recent studies have suggested that ferroptosis is involved in the process of CVC [32]. Cell ferroptosis involves lipid peroxidation as the core process and iron and ROS as the key points. Morphology of the cell nucleus remains unchanged, and the main manifestations include mitochondrial shrinkage, membrane density increase, and outer membrane rupture, resulting in cell apoptosis and pyrodeath. The release of many proinflammatory factors leads to endothelial cell injury, enhanced oxidative stress response, and proliferation and transdifferentiation of VSMCs, thereby promoting CVC [33]. It has been suggested that inhibition of the solute carrier family 7 member 11 (SLC7A11)/glutathione (GSH)/glutathione peroxidase 4 (Gpx4) axis can promote VC under CKD conditions. Proinflammatory macrophages interact with Gpx4 to promote ferroptosis. High phosphorus induces the decrease in VSMCs viability, the increase in ptgs2mRNA level, the decrease in GSH content, and the increase in lipid peroxidation and ROS level, induces ferroptosis of cells, and promotes osteogenic transdifferentiation. Ferrostatin 1, a ferroptosis inhibitor, can effectively reduce the levels of ROS and lipid peroxidation and alleviate the development of calcification [34]. The results of in vitro culture of rat and human arterial rings have shown that ferrostatin 1 can reduce high phosphorus-induced arterial ring calcification, suggesting that ferroptosis is involved in the regulatory process of VC. In vivo studies have suggested that treatment with ferroptosis inhibitor in 5/6 nephrectomy-induced aortic calcification rat models of CKD can significantly improve aortic calcification, suggesting that inhibiting ferroptosis can improve arterial calcification in CKD rats [35].

### 3.3. Extracellular Vesicles

Extracellular vesicles (EVs) are mainly released by the cells into their surrounding environment and contain cell-derived biomolecules, such as proteins and nucleic acids. They regulate intercellular information transfer and function in all aspects of cell biology [36]. One of the most important EV types are exosomes. A number of studies have shown that inflammatory oxidative stress in CKD promotes the release of EVs from dedifferentiated VSMCs mediating Ca^2+^ entrance into the cytoplasm, thereby inducing osteogenic phenotypic transdifferentiation of VSMCs [37]. Sox9 promotes the secretion of EVs containing pro-calcification factors and induces VC. When exosomes derived from VSMCs in a CVC mouse model are added to VSMCs of the control group, the control group cells start expressing proteins related to calcification, which suggests that exosomes act as the vehicle for signals between VSMCs [38]. Exosomes can also regulate VC by activating inflammatory pathways and immune responses, and by promoting autophagy in VSMCs. After EVs intervene in VSMCs, phosphate transporter PiT-1 is upregulated, which causes calcification, with increased levels of Osterix, Runx2, and osteocalcin, and decreased levels of SM22α. In contrast, knockdown of PiT-1 inhibits CVC [39]. The expression of STAT1 is upregulated in exosomes after high-phosphorus stimulation, inducing CVC in VSMCs. The use of fludarabine to inhibit STAT1 expression in VSMCs or the use of small interfering RNA to knock down STAT1 expression alleviates calcification of VSMCs. High phosphorus-induced EVs in the serum of CKD rats promote VSMCs calcium salt deposition, and GW4869 inhibits systemic EVs’ biosynthesis to improve aortic calcification in CKD mice [40]. All of these facts suggest that EVs are immensely involved in VC. In recent years, the research on exosomes has expanded, which may provide a potential research direction for improving CVC.

### 3.4. MicroRNA (miRNA)

Recently, miRNAs have been suggested to have a bidirectional regulatory function in osteogenic transformation and calcification of VSMCs. miRNAs are a class of noncoding RNAs that affect post-transcriptional regulation. They are enriched in the exosomes of mice with CVC in CKD. Inhibition of endogenous miRNA-125b promotes osteogenic differentiation of VSMCs by targeting Osterix, whereas exosomes rich in miRNA-204/miRNA-211 reduce VC of VSMCs by targeting BMP2. miRNA-26 negatively regulates osteogenic transdifferentiation of SMCs via the Wnt/β-catenin pathway [41]. Research has found that the level of serum miR-126 in patients with CKD are reduced, which regulates endothelial dysfunction. It is associated with uremic toxins and affects vascular physiology by disrupting endothelial, platelet, and immune functions, thereby increasing the risk of CVD, especially CVC in CKD patients [42]. High phosphorus levels stimulate ECs to secrete miRNA-670-3p-rich exosomes, promoting VC and deposition of mineralized nodules. Knockdown of miRNA-670-3p in ECs exosomes weakens the expression of calcification indicators of VSMCs. Insulin-like growth factor 1 (IGF-1) is the target gene directly bound to miR-670-3p, and overexpression of IGF-1 can reverse the pro-calcification effect of miRNA-670-3p. Endogenous miRNA-205 acts on Runx2 and Smad1 to inhibit the transduction of the osteogenic phenotype of SMCs [43]. Studies conducted by our research group have shown that miRNA-214 can promote osteogenic transdifferentiation of SMCs through PTEN in vivo and in vitro [44]. MiRNAs also play an important role in aging and calcification of VSMCs and play a critical role in CVC. They have the opposite functions, promoting and inhibiting CVC. Further research into this field may provide potential therapeutic targets to postpone CVC in patients with CKD.

### 3.5. Long Noncoding RNA (lncRNA)

lncRNA is a noncoding RNA, which contains greater than 200 nucleotides. The complex and precise regulatory functions played by lncRNAs in development and gene expression include epigenetics, cell differentiation, glucose and lipid metabolism, and many other life activities. Research has shown that lncRNAs are associated with the pathophysiology of kidney disease and contributes greatly to CVC [45]. The osteogenic differentiation of VSMCs induced by high phosphorus may be regulated by several lncRNAs, such as the lncRNA NONHSAT058810.2, lncRNA NONHSAT197162.1, lncRNA NONHSAT033640.2, lncRNA NONHSAT036152.2, lncRNA NONHSAT179247.1, lncRNA NONHSAT162315.1, lncRNA NONHSAT061050.2, and lncRNA NONHSAT006046.2 [46,47]. LncRNA H19 increases Runx-2 protein and inhibits SM22-α and α-SMA expression, thereby promoting CVC [48]. The engineered exosome-derived lncRNA ANCR effectively targets SMC for rapid reconstruction and significantly inhibits differentiation of osteoblast-like cells, antagonizing CVC caused in CKD [49]. The lncRNA FAS-AS1 is associated with the development of ESRD and the occurrence of CVC. FAS-AS1 is upregulated in patients with CVC, especially those with severe calcification. Calcified VSMCs show significantly increased levels of Ca^2+^, interleukin-6 (IL-6) and reactive oxygen species (ROS), which are attenuated by silencing FAS-AS1. Knockout of FAS-AS1 inhibits hyperphosphatemia induced VC via reducing oxidative stress and inflammation [50]. LncRNAs play a key role in the process of CVC. Treatments targeting lncRNAs may help improve this pathology.

### 3.6. Aging

Recent studies have shown that a variety of pathological factors, such as inflammation, oxidative stress damage, and uremic toxins, can induce premature aging of VSMCs in the CKD state [51]. Aging is closely related to osteogenic transdifferentiation of VSMCs and CVC. Aging VSMCs are more prone to osteogenic transformation. The expression of aging-related markers is upregulated, and the expression of Runx2, ALP, BMP2, and other osteogenic markers is increased [52]. Clinical studies have demonstrated an increasing number of cells positive for p16 and β-galactinase-related markers of aging in the coronary media and increased expression of Runx2 in patients with CKD, that is, the co-phenotype of vascular aging and VC [53]. In vivo experiments have shown that after uremic toxin stimulation, the expression levels of p53, the age-related marker of VSMCs in the rat aortic arch, and the precursor of lamin A are upregulated, which can activate the DNA damage response, increase β-galactosidase activity, promote VSMCs’ aging and osteogenic differentiation, and cause aortic calcification [54]. Aging may have a more complicated mechanism in the process of CVC, and the existing research is still in an early stage. Additional research is needed to probe into all aspects of CVC.

## 4. Signaling Pathways of CVC

### 4.1. Wnt/β-Catenin

The Wnt/β-catenin signaling pathway affects body homeostasis. The Wnt/β-catenin pathway promotes CVC via different mechanisms. The classical Wnt/β-catenin pathway mechanism is to bind extracellular Wnt ligands to the seven-channel transmembrane coiled receptor to activate β-catenin. It binds to transcription factors to form transcription complexes and activates subsequent targets. When calcification occurs, CVC markers are upregulated [55]. The non-classical Wnt/β-catenin pathway activates two branches for the pathological calcification pathway via Ror1/2 coreceptors, namely the Wnt/Ca^2+^ and planar cell polarity (PCP) pathway [56], triggering activation of small GTPasesRho, leading to cytoskeletal changes and lateral asymmetry. This leads to the transcriptional expression of downstream calcification signals.

Studies conducted by our research team show that high phosphorus/high PTH can stimulate ECs to undergo endothelial-to-mesenchymal transition (EndMT), obtaining the phenotype of mesenchymal stem cells and differentiating into osteoblasts under the corresponding induction environment, thereby promoting the development of CVC in CKD [57]. Wnt/β-catenin contributes to VC induced by a hyperphysiological dose of high phosphorus/high PTH, and inhibiting the activation of this signaling pathway by applying β-catenin siRNA or Wnt inhibitor Dickkopf-1 (DKK1) can block the progression of VC [58]. The Wnt/β-catenin signaling pathway promotes CVC. There are also some therapies for targeting the Wnt/β-catenin pathway. It is hoped that clinical trials can be put into research in the future.

### 4.2. Notch

Notch signaling plays an important role in the body. It regulates transcription of Runx2. The Notch signaling pathway upregulates the success-related Msx2 gene in VSMCs [59]. Multiple omics studies have shown that tissue-specific EVs are associated with pro-calcitrogenic Notch and Wnt signaling in the carotid artery and aortic valve, respectively. Our study has shown that by regulating miRNA-29a to upregulate GSAP expression, PTH can activate the Notch1 pathway, leading to endothelial-to-mesenchymal transition (EndMT). However, inhibition of EndMT by gamma secretase activating protein (gSAP) can partially alleviate CVC in CKD [60]. However, Notch also has an inhibitory effect on VC. Usp9xs-nitroso-induced Notch1 activation inhibits nitric oxide (NO)-regulated calcification of porcine aortic VC. Wnt activates Notch and counteracts Notch3 inhibition. It results in the prevention of chondrogenesis of VSMCs [61]. In addition, Notch3 derived from exosomes released by ECs reduces CVC by inhibiting mTOR when present in a high-glucose environment. Taken together, previous studies have provided important information for the Notch signaling pathway in CVC. It is clear that the Notch signaling pathway can accelerate the development of CVC by complex cascade reactions.

### 4.3. Runx2/BMP

In a mouse model of CKD, SIRT6-HMGB1 deacetylation can inhibit the transport of HMGB1 from the nucleus to the cytoplasm and induce Runx2, OPN, and muscle segment homeobox 2 (Msx2) expression, thereby regulating CVC in CKD [62]. In addition to inducing the transcription of osteogenic genes, Runx2 can also respond to DNA damage through histone H2AX phosphorylation, acting as a bridge between DNA damage signals and osteogenic gene transcription and cell apoptosis, thereby accelerating CVC. Attention was initially garnered on the role of BMPs in promoting heterotopic bone formation. The osteogenic signal of BMP2 is significantly enhanced in dedifferentiated VSMCs. The expression of osteogenic transcription factors Runx2 and Msx2 can be enhanced via the ALK/Smad pathway to promote ALP expression and matrix mineralization [63]. Lack of BMP2/4 restrains matrix gamma carboxyl glutamic acid protein (MGP), which can also result in VSMCs’ osteogenic differentiation and mineralization. In contrast, selective BMP inhibitors can downregulate the osteogenic differentiation of VSMCs and inhibit arterial media calcification [64]. The oxidative stress of CKD leads to the accumulation of ROS in vivo and increases Runx2 level via the protein kinase signaling pathway, thereby promoting phenotypic transformation of VSMCs [65]. BMP2 accelerates the calcification of VSMCs, whereas inhibition of BMPs signaling reduces osteogenic differentiation. The Runx2/BMP signaling pathway can affect CVC at the level of signal transduction. Clearly, more research is needed to clarify the cellular interactions of the Runx2/BMP signaling pathway.

### 4.4. STAT

Signal transduction and transcriptional activation factor (STAT) is a transcription factor, and extracellular stimuli, such as Janus kinase (JAK), can activate STAT. Activated STAT regulates the expression of target genes [66]. Patients with STAT1 mutations may have significant CVC. Previous studies have shown that adding monocyte-expressed urokinase to VSMCs can activate STAT1 and inhibit the proliferation of VSMCs [67]. STAT1 can upregulate RUNX2 and BMP2, thus promoting the occurrence of vascular atherosclerosis and VC in mice [68]. After the intervention of STAT1 in VSMCs, the generation and activation of STAT1 are promoted, and VSMCs lose their contractile phenotype and transdifferentiate into the osteoblast-like phenotype. In mice, STAT1 knockout can activate the FGF pathway in osteoblasts, thereby promoting osteogenic differentiation. STAT3 contributes to the transformation in VSMCs by tumor suppressor protein M. As a result, erythropotropin-mediated calcification occurs in rats. Silencing of STAT3 inhibits the expression and mineralization of genes related to osteogenic transformation of human SMCs induced by tumor suppressor protein M [69]. The above evidence suggests that the STAT signaling pathway promotes CVC; however, further studies are needed to fully elucidate the mechanisms in CVC.

## 5. Conclusions and Future Directions

In summary, CVC has a high incidence and poor prognosis in patients with CKD. Current studies suggest that CVC is a process regulated by multiple factors, mechanisms, and signals, which are independent and interrelated. Based on the mechanisms, a series of clinical trials for the therapy of CVC, such as hemodialysis, drugs including calcium and phosphorus preparations, vitamin D, and sodium thiosulfate, which have attracted attention in recent years, are being actively carried out. However, there is currently no method to inhibit CVC and restore vascular compliance. It is essential to conduct more in-depth basic research on the pathogenesis of CVC so as to find effective means to prevent and treat CVC in CKD.
